# Acute and chronic cardiac adaptations in adults born preterm

**DOI:** 10.1113/EP089917

**Published:** 2022-03-09

**Authors:** Adam J. Lewandowski

**Affiliations:** ^1^ Oxford Cardiovascular Clinical Research Facility Division of Cardiovascular Medicine Radcliffe Department of Medicine University of Oxford Oxford UK

**Keywords:** cardiac physiology, cardiac remodelling, cardiovascular risk, prematurity, preterm birth

## Abstract

**New Findings:**

- **What is the topic of this review?**
  Studies using cardiovascular magnetic resonance imaging and echocardiography to investigate cardiac alterations at rest and during exercise‐induced physiological stress in adults born preterm.
- **What advances does it highlight?**
  People born preterm have a greater long‐term cardiovascular risk, which may be explained in part by their cardiac structural and functional alterations. They have potentially adverse alterations in left and right ventricular structure and function that worsens with blood pressure elevation; an impaired myocardial functional reserve; and an increase in diffuse myocardial fibrosis that may drive their lower diastolic function.

**Abstract:**

Preterm birth accounts for more than 10% of births worldwide and associates with a long‐term increase in cardiovascular disease risk. The period around preterm birth is a rapid and critical phase of cardiovascular development, which might explain why changes in multiple components of the cardiovascular system have been observed in individuals born preterm. These alterations include reduced microvascular density, increased macrovascular stiffness, and higher systolic and diastolic blood pressure. Cardiac alterations have been observed in people born preterm as early as neonatal life and infancy, with potentially adverse changes in both left and right ventricular structure and function extending into adulthood. Indeed, studies using cardiovascular magnetic resonance imaging and echocardiography have demonstrated that preterm‐born individuals have structural cardiac changes and functional impairments. Furthermore, myocardial tissue characterization by cardiovascular magnetic resonance imaging has demonstrated an increase in left ventricular diffuse myocardial fibrosis in young adults born preterm, and under acute physiological stress, their myocardial functional reserve assessed by echocardiography is reduced. The preterm heart is also more susceptible to chronic systolic blood pressure elevation, with a significantly greater increase in left ventricular mass as systolic blood pressure rises observed in preterm‐born compared to term‐born young adults. Given these known, potentially adverse acute and chronic cardiac adaptations in the preterm‐born population, primary prevention strategies are needed to reduce long‐term cardiovascular disease risk in this subgroup of the population.

## 1 INTRODUCTION

Preterm birth (<37 weeks’ gestation) is a common birth complication that affects 1 in 10 births worldwide (Chawanpaiboon et al., 2019). Due to advances in perinatal clinical care, survival rates greater than 95% are often achieved across the gestational age range of the preterm population. Nevertheless, preterm birth remains a leading cause of neonatal morbidity and mortality globally. Further to this, mounting evidence demonstrates that being born preterm has long‐term cardiovascular consequences, with large‐scale birth registry studies showing that preterm birth is associated with an increased risk of hypertension (Crump et al., 2011) and ischaemic heart disease in young adulthood (Crump et al., 2019); heart failure in childhood, adolescence and young adulthood (Carr et al., 2017; Crump et al., 2021); and early cardiovascular‐related mortality (Risnes et al., 2021). As a result, observational cohort studies in people born preterm have investigated structural and functional adaptations of the cardiovascular system, including cardiac remodelling (Lewandowski et al., 2020).

## 2 CHANGES IN CARDIAC STRUCTURE AND FUNCTION IN YOUNG ADULTS BORN PRETERM

In 2013, the first comprehensive study investigating the association between preterm birth and left ventricular (LV) remodelling in young adulthood was published (Lewandowski et al., 2013). Using cardiovascular magnetic resonance (CMR) imaging and the creation of a unique cardiac statistical atlas from the CMR images, 102 young adults (mean age 25 years) born preterm were studied and compared using the same investigations with a group of 102 age and sex matched young adults born at term and 30 sex matched adults born at term who were 10 years older. This latter group was included to assess normal differences related to cardiovascular ageing. Preterm‐born young adults were shown to have smaller LV volumes with shorter LV lengths, greater LV mass and impaired LV longitudinal systolic strain. These latter impairments were most pronounced in those born preterm to hypertensive pregnancies, which is an independent predictor of long‐term cardiovascular risk (Frost et al., 2021). In addition, LV longitudinal diastolic strain rate was impaired in the preterm‐born young adults compared to age and sex matched controls but was similar to the group of term‐born adults who were 10 years older. The greatest predictor of the remodelling of the LV was the degree of prematurity.

In a follow‐on paper studying the same cohort, it was demonstrated that young adults born preterm had smaller right ventricular (RV) volumes and greater RV mass compared to both age and sex matched preterm‐born young adults and sex matched term‐born adults who were a decade older (Lewandowski et al., 2013). Systolic volumetric functional impairments were more pronounced for the RV, with a significantly lower RV ejection fraction, including a proportion of the cohort (6%) with measures below the normal clinical range. As with the LV, the strongest predictor of the RV changes was the degree of prematurity, though changes were more pronounced for the RV than the LV. Indeed, for each week decrease in gestational age in the preterm group, there was a 2.74% relatively greater RV mass in young adulthood compared to a 1.47% relatively greater LV mass. For ejection fraction, there was a significant 2.51% relatively lower RV ejection fraction for each week‐shorter gestation, though there was no change in LV ejection fraction. Although pulmonary function was not assessed in this cohort, days of postnatal ventilation accounted for some of the variation in RV remodelling. Therefore, further work was carried out to investigate whether these changes were caused by alterations in pulmonary physiology that have been observed in other preterm‐born adult studies.

## 3 RIGHT VENTRICULAR CHANGES OUT OF PROPORTION TO ALTERATIONS IN PULMONARY PHYSIOLOGY

In an additional cohort of 101 young adults, of which 54 were born preterm and 47 were born at term, echocardiography and CMR were used in combination with spirometry measures to assess the relationship between RV and pulmonary physiology (Mohamed et al., 2020). It was demonstrated that young adults born preterm had smaller RV end‐diastolic areas and fractional area of change by echocardiography compared to their term‐born peers, with similarly lesser RV volumes and ejection fraction by CMR. CMR analyses also revealed greater RV mass in the preterm group, as previously shown. Interestingly, although echocardiography scans revealed greater pulmonary vascular resistance in the preterm‐born young adults, the RV remained coupled to its pulmonary physiology.

## 4 CARDIAC ALTERATIONS EXTEND FROM BIRTH INTO ADULTHOOD

Studies in both animals and humans have demonstrated the relevance of the preterm postnatal window to the emergence of these cardiac alterations. However, many of the human studies have been relatively small‐scale, with measurements done at a single time point. To determine whether similar cardiac alterations first observed during neonatal life and infancy extend into adulthood, a systematic review and meta‐analysis of the literature, including both echocardiography and CMR studies, was performed (Telles et al., 2020). A total of 38 publications comparing cardiac phenotype in uncomplicated, asymptomatic preterm cases (<37 weeks’ gestation) to age‐matched term‐born controls were included, of which 32 were unique observational studies. The results demonstrated that preterm‐born individuals have smaller ventricular dimensions from birth to young adulthood; lower LV diastolic function across all developmental stages; lower RV systolic function across all developmental stages that worsens with ageing; and an accelerated rate of LV hypertrophy from childhood to young adulthood.

## 5 RISK OF EARLY HEART FAILURE AND A REDUCTION IN MYOCARDIAL FUNCTIONAL RESERVE

One of the most striking findings in the first registry‐based study showing an association between being born preterm and the risk of early heart failure in childhood and adolescence was the magnitude of the risk. Indeed, gestational age was inversely associated with a greater risk of heart failure, with adjusted incidence relative risks for heart failure up to 17‐fold greater for those born at <28 weeks’ gestation (extremely preterm) compared to those born at term (Carr et al., 2017). Nevertheless, up to two‐thirds of children and young adults recover their cardiac function after the episode, and the majority of individuals with heart failure in the registry‐based study were actually born at term (Carr et al., 2017). This suggests that a form of pathogen or familial predisposition is still the most likely trigger of heart failure across this age group (Leeson & Lewandowski, 2017). However, we hypothesised that the underlying alterations in cardiac remodelling that emerge during the early preterm postnatal window may result in a reduction in myocardial functional reserve, resulting in a more rapid decline in cardiac function with greater susceptibility to acute events such as myocarditis in childhood or severe hypertension in adulthood.

**FIGURE 1:**
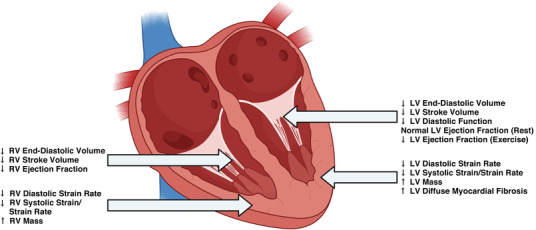
Observed cardiac structural and functional changes in young adults born preterm

To test this hypothesis, 54 young adults born preterm were compared with 47 young adults born at term to determine whether they had an impaired LV response to acute exercise stress (Huckstep et al., 2018). Maximal cardiopulmonary exercise testing was first used to determine peak power, followed by submaximal exercise testing at 40% (mild), 60% (moderate) and 80% (high) of peak exercise intensity. At each stage, echocardiography imaging was performed to assess LV function. As previously shown by other studies, there was no difference between groups for LV ejection fraction at rest, but by moderate and high intensity exercise, LV ejection fraction was 7% lower in the preterm‐born than term‐born young adults (Figure 1). These results are suggestive of a reduced myocardial functional reserve in people born preterm. In addition, these reductions were greatest as gestational age decreased and associated with resting measures of LV length and longitudinal systolic strain, which were both lower in the preterm‐born than term‐born group.

It was also investigated whether the reduced myocardial functional reserve may predict maximal exercise capacity and recovery in this cohort, which have been shown by other studies to be impaired (Lewandowski et al., 2020). The change in LV ejection fraction from rest to mild and moderate exercise intensity was shown to explain a significant proportion of the lower maximal oxygen uptake and slower heart rate recovery in the preterm‐born young adults (Huckstep et al., 2021), providing further evidence that cardiac alterations are relevant to cardiopulmonary physiology and capacity.

## 6 LEFT VENTRICULAR RESPONSE TO SYSTEMIC BLOOD PRESSURE ELEVATION

Given that preterm‐born adults are at an increased risk of developing hypertension and are more likely to have greater blood pressure levels than their term‐born peers as early as childhood (Lewandowski et al., 2020), our group has investigated what effect this may have on the observed changes in the LV of young adults born preterm. In a cohort of 468 individuals with CMR imaging, of which 200 were born preterm, it was shown that there was a significant, positive association in both preterm‐ and term‐born young adults between systolic blood pressure and LV mass (Mohamed et al., 2021). In addition to a leftward shift in the regression lines in those born preterm compared to those born at term, there was a stronger association between systolic blood pressure and LV mass in the preterm‐born compared to term‐born young adults. Indeed, there was a 2.0‐fold greater increase in LV mass per 1 mmHg systolic blood pressure elevation in the preterm group compared to the term‐born group. This difference was particularly significant in the young adults born <28 weeks’ gestation, with a 2.5‐fold greater LV mass increase per 1 mmHg elevation in systolic blood pressure compared to those born at term. These results suggest that secondary insults, such as blood pressure elevation, may have a greater impact on the remodelled preterm heart. As such, earlier interventions to reduce blood pressure elevation and to target improvements in cardiac structure and function are needed in this population group.

## 7 INCREASED DIFFUSE MYOCARDIAL FIBROSIS UNDERLIES LEFT VENTRICULAR DIASTOLIC FUNCTIONAL IMPAIRMENTS

In both sheep and rat models of preterm birth, it has been demonstrated that the cardiomyocytes have accelerated hypertrophy and that there is an increase in interstitial myocardial collagen (Bensley et al., 2010; Bertagnolli et al., 2014), which relates to reduced systolic and diastolic function measures assessed using echocardiography. We therefore sought to determine whether young adults born preterm have an increase in LV myocardial fibrosis. We used CMR imaging to non‐invasively assess focal myocardial fibrosis in the left ventricle with late gadolinium enhancement and LV diffuse myocardial fibrosis by pre‐ and postcontrast T1 mapping (Lewandowski et al., 2021). In addition, echocardiography and CMR were used to assess LV systolic and diastolic function. Preterm‐born young adults had greater LV diffuse fibrosis, but no change in focal fibrosis, compared to those born at term. This elevation in LV diffuse myocardial fibrosis was strongly correlated with impaired LV diastolic function measured by both echocardiography and CMR, and was also shown to mediate the relationship between the degree of prematurity and the extent of LV diastolic functional impairment.

## 8 POTENTIAL TARGETS FOR INTERVENTION

Despite observational and epidemiological studies identifying people born preterm having unique alterations in cardiac physiology and cardiovascular disease risk, intervention strategies for this subgroup of the population have been limited. The postnatal window may offer an opportunity for intervention given its importance for cardiac remodelling. CMR imaging in preterm‐born young adults who had been randomized at birth to different milk feeding diets showed that those who were randomized to an exclusive human milk diet postnatally (*n* = 30) had greater LV and RV volumes and systolic function than those that were randomized to an exclusive formula diet (*n* = 16) (Lewandowski et al., 2016). More recently, in a study of 80 preterm infants followed at multiple time points for echocardiography scans during the first year of life (32 weeks postmenstrual age; 36 weeks postmenstrual age; and 1 year corrected age), it was shown that, for each additional week of exposure to mother's own milk, there was an improvement in LV and RV function, as well as lower pulmonary vascular resistance by 1 year corrected age (El‐Khuffash et al., 2021). Furthermore, the preterm infants exposed to higher levels of mother's own milk (>28 days) had similar cardiac and pulmonary function measures by 1 year corrected age compared to a group of 100 age and sex matched term‐born infants. These two studies highlight the importance and potential benefit of human milk for short‐term and long‐term cardiopulmonary performance in people born preterm, which may be mediated through several possible mechanisms (El‐Khuffash et al., 2020). Similar benefits associated with breastfeeding to both cardiac and vascular structure and function have been observed in children who were low birth weight due to fetal growth restriction (Rodriguez‐Lopez et al., 2016). Further work is needed to identify the optimal preterm infant nutrition, taking into consideration feasibility at both the individual and population level across healthcare systems and settings. It is also possible that targeting lifestyle during adolescence and young adulthood could alter the trajectory of cardiovascular risk in the preterm population with ageing. For instance, exercise training may preferentially remodel the cardiopulmonary system, though this remains to be determined (Williamson et al., 2018).

## 9 CONCLUSIONS

In conclusion, preterm birth associates with a unique cardiac phenotype that may put these individuals at increased risk of cardiovascular disease. The number of people born preterm each year continues to rise, as does the percentage of individuals surviving. While much is known about the immediate costs and pressures preterm birth places on healthcare systems, little is known about this in the longer term. Primary prevention strategies to reduce risk in this group are needed and, in order to be implemented at scale, will require input from multi‐disciplinary teams including the affected patients.

## COMPETING INTERESTS

None.
